# Influence of oncogenic mutations and tumor microenvironment alterations on extranodal invasion in diffuse large B‐cell lymphoma

**DOI:** 10.1002/ctm2.221

**Published:** 2020-11-24

**Authors:** Rong Shen, Peng‐Peng Xu, Nan Wang, Hong‐Mei Yi, Lei Dong, Di Fu, Jin‐Yan Huang, Heng‐Ye Huang, Anne Janin, Shu Cheng, Li Wang, Wei‐Li Zhao

**Affiliations:** ^1^ State Key Laboratory of Medical Genomics National Research Center for Translational Medicine at Shanghai Shanghai Institute of Hematology Ruijin Hospital Affiliated to Shanghai Jiao Tong University School of Medicine Shanghai China; ^2^ Department of Pathology Shanghai Ruijin Hospital Shanghai Jiao Tong University School of Medicine Shanghai China; ^3^ School of Public Health Shanghai Jiao Tong University School of Medicine Shanghai China; ^4^ Inserm Hôpital Saint Louis Université Paris 7 Paris France; ^5^ Laboratory of Molecular Pathology Pôle de Recherches Sino‐Français en Science du Vivant et Génomique Shanghai China

**Keywords:** diffuse large B‐cell lymphoma, extracellular matrix, extranodal invasion, MYD88, oncogenic mutation, regulatory T cells, tumor microenvironment

## Abstract

**Background:**

Diffuse large B‐cell lymphoma (DLBCL) is an aggressive subtype of lymphoma, and multiple extranodal involvement (ENI) indicates adverse clinical outcomes. The aim of this study was to investigate the influence of oncogenic mutations and tumor microenvironment alterations on ENI in DLBCL.

**Methods:**

The clinical features of 1960 patients with newly diagnosed DLBCL were analyzed, and DNA and RNA sequencing was performed on 670 and 349 patients, respectively. Oncogenic mutations and tumor microenvironment alterations were compared according to ENI and evaluated in zebrafish patient‐derived tumor xenograft models.

**Results:**

Multiple ENI was significantly associated with poor performance status, advanced stage, elevated serum lactate dehydrogenase, low response rate, and inferior prognosis. Lymphoma invasion of the bones, spleen, bone marrow, liver, and central nervous system were independent unfavorable prognostic factors. *MYD88* was frequently mutated in patients with multiple ENI, co‐occurred with mutations in *CD79B*, *PIM1*, *TBL1XR1*, *BTG1*, *MPEG1*, and *PRDM1*, and correlated with invasion of the bones, kidney/adrenal glands, breasts, testes, skin, and uterus/ovaries. For tumor microenvironment alterations, patients with multiple ENI showed higher regulatory T‐cell (Treg)‐recruiting activity, but lower extracellular matrix‐encoding gene expression, than those without ENI and with single ENI. Elevated Treg‐recruiting activity was related to mutations in *B2M*, *SGK1*, *FOXO1*, *HIST1H1E*, and *ARID1A*, and correlated with invasion of the bone marrow and thyroid. Additionally, mutations in *MYD88*, *PIM1*, *TBL1XR1*, *SGK1*, *FOXO1*, *HIST1H1E*, and *ARID1A* were associated with decreased major histocompatibility complex class I expression. Zebrafish models further revealed relationships between *MYD88* mutations and invasion of the kidneys and gonads, as well as *B2M* mutations and invasion of the bone marrow. Increased CXCR4 expression is linked to bone marrow invasion in an organotropic way.

**Conclusions:**

Our findings thus contribute to an improved understanding of the biological behavior of multiple ENI and provide a clinical rationale for targeting ENI in DLBCL.

## Graphical abstract

1. DLBCL patients with multiple extranodal involvement presented advanced disease stage and poor prognosis.

2. Oncogenic mutations were associated with multiple extranodal involvement.

3. Increased Treg‐recruiting activity and decreased extracellular matrix expression of the tumor microenvironment correlated with multiple extranodal involvement.

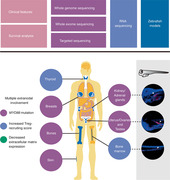

**Abbreviations**
CHT: caudal hematopoietic tissue
CNS: central nervous system
CT: computed tomography
DLBCL: diffuse large B‐cell lymphoma
ECM: extracellular matrix
ECOG: Eastern Cooperative Oncology Group
ENI: extranodal involvement
FFPE: formalin‐fixed paraffin‐embedded
GSEA: gene set enrichment analysis
IPI: International Prognostic Index
LDH: lactate dehydrogenase
MDSC: myeloid‐derived suppressor cell
MRI: magnetic resonance imaging
NCCN: National Comprehensive Cancer Network
NF‐κB: nuclear factor kappa‐B
non‐GCB: non‐germinal center B cell
OS: overall survival
PET‐CT: positron emission tomography‐computed tomography
PFS: progression‐free survival
R‐CHOP: rituximab, cyclophosphamide, doxorubicin, vincristine, and prednisone
SNV: single nucleotide variation
SPSS: Statistical Package for the Social Sciences.
Th2: T helper 2
TIP: tumor immunophenotyping
Treg: regulatory T
WES: whole exome sequencing
WGS: whole genome sequencing
WHO: World Health Organization

## 1 INTRODUCTION

Diffuse large B‐cell lymphoma (DLBCL) is the most common histological subtype of aggressive non‐Hodgkin lymphoma and represents a heterogeneous entity.^1^ Approximately one‐third of DLBCL arises primarily from extranodal sites, most frequently the gastrointestinal (GI) tract, breasts, testes, thyroid, skin, and uterus/ovaries,^2^ or originates from the lymph nodes and spreads secondarily to extranodal organs, including the bones, spleen, bone marrow, kidney/adrenal glands, lungs, and liver.^3^ Multiple extranodal involvement (ENI) is an important factor of the International Prognostic Index (IPI) and indicates poor prognosis of patients treated by rituximab in combination with cyclophosphamide, doxorubicin, vincristine, and prednisone (R‐CHOP).^4^, ^5^ More recently, the National Comprehensive Cancer Network database (NCCN)‐IPI has been developed and identifies the involvement of the bone marrow, central nervous system (CNS), liver/GI tract, and lungs as unfavorable risk factors.^6^ Therefore, it remains critical to define the underlying molecular features, particularly oncogenic mutations and tumor microenvironment alterations, which contribute to ENI in DLBCL.

Accumulating data suggest the occurrence of divergent biological behaviors in extranodal DLBCL. For instance, DLBCL in immune‐privileged sites, including the CNS and testes, as well as breast, uterine, and cutaneous DLBCL, are related to prevalent non‐germinal center B cell (non‐GCB) phenotype and *MYD88*/*CD79B*‐mutated genotype. In contrast, gastric and thyroid DLBCL lack *MYD88* mutations.^7^ Other gene mutations related to *MYD88*/*CD79B* mutations, including *PIM1*, *BTG1*, *TBL1XR1*, *MPEG1*, and *PRDM1*, were frequently acquired in breast, testis, and cutaneous lymphoma.^8^ In addition to malignant lymphocytes themselves, the tumor microenvironment has emerged as another major predictor of lymphoma progression.^9^ Immune cells, extracellular matrix (ECM), and endothelial cells are important microenvironmental components in DLBCL.^10^ Variable immune cells, including regulatory T (Treg) cells, T helper 2 (Th2) cells, and myeloid‐derived suppressor cells (MDSCs), have been reported to promote immune evasion and tumor dissemination.^10^, ^11^ Stromal elements have also shown prognostic significance, as the expression of genes encoding the ECM and endothelial cells leads to tumor progression in R‐CHOP‐treated DLBCL patients.^12^ The loss of major histocompatibility complex (MHC) class I results in impaired antigen presentation and deficient immunological recognition in the tumor microenvironment. Chemokine receptors are implicated in tumor cell migration and directly induce organ‐specific invasion.^13^ However, their contributions to organotropic invasion in DLBCL need to be further investigated.

In the present study, we investigated the clinical characteristics and prognostic significance of multiple ENI in a large cohort of 1960 patients with newly diagnosed DLBCL, and performed genomic and transcriptomic analyses to illustrate the oncogenic mutations and tumor microenvironment alterations associated with ENI.

## 2 PATIENTS AND METHODS

### 2.1 Patients

**FIGURE 1:**
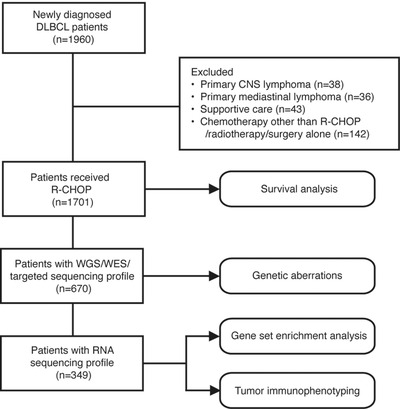
Flowchart of the patient selection and methods Abbreviations: DLBCL, diffuse large B‐cell lymphoma; CNS, central nervous system; R‐CHOP, rituximab, cyclophosphamide, doxorubicin, vincristine and prednisone; WES, whole exome sequencing; WGS, whole genome sequencing.

A flow chart is outlined in Figure 1 to describe the patient selection. From September 2002 to August 2019, 1960 patients with newly diagnosed DLBCL based on registry data were included, with the last follow‐up through February 1, 2020. Histological diagnosis was reviewed and confirmed by two experienced pathologists (Hong‐Mei Yi and Lei Dong) based on World Health Organization (WHO) classification.^1^ Survival analysis was performed on 1701 patients who received R‐CHOP, excluding patients with primary CNS lymphoma (n = 38), primary mediastinal lymphoma (n = 36), supportive care (n = 43), or chemotherapy other than R‐CHOP, radiotherapy, or surgery alone (n = 142). DNA and RNA sequencing were performed on 670 and 349 patients with available tumor and blood samples, respectively, for detection of genetic aberrations, gene set enrichment analysis (GSEA), and tumor immunophenotyping (TIP). The study was approved by the Shanghai Ruijin Hospital Review Board, and informed consent was obtained in accordance with the Declaration of Helsinki.

Clinical data included age, gender, Eastern Cooperative Oncology Group (ECOG) performance status, Ann Arbor stage, serum lactate dehydrogenase (LDH), and specific sites of ENI.^14^ Fifteen extranodal sites were evaluated in this study: nasal cavity, thyroid, breasts, lungs, GI tract, liver, pancreas, spleen, kidney/adrenal glands, testes, uterus/ovaries, bones, bone marrow, skin, and CNS.^2^, ^3^ Sites of ENI were determined by approaches available: first by histology, and then by radiology including positron emission tomography‐computed tomography (PET‐CT), thoraco‐abdominal CT, or magnetic resonance imaging (MRI),^15^ in a hierarchical order. Bone marrow involvement was histologically established by bone marrow biopsy.^16^

### 2.2 DNA sequencing

Genomic DNA was extracted from frozen tumor tissue by a QIAamp DNA Mini Kit (Qiagen, Hilden, Germany), or from formalin‐fixed paraffin‐embedded (FFPE) tumor tissue by a GeneRead DNA FFPE Tissue Kit (Qiagen), based on the manufacturer's guidelines. For 216 patients, whole‐exome sequencing (WES) was carried out on frozen tumor tissue (n = 132), or on FFPE tumor tissue quality controlled by agarose gel electrophoresis (n = 84). For 109 patients, whole‐genome sequencing (WGS) was performed on frozen tumor tissue. WES (n = 25, divided into five groups) and WGS (n = 17) were performed on 42 matched peripheral blood samples randomly selected to build a somatic mutation calling principle and to exclude germ‐line polymorphisms. Three hundred forty‐five patients with FFPE tumor tissue were analyzed by targeted sequencing of 55 lymphoma‐associated genes. Genome Analysis Toolkit (GATK, v3.7.0), Haplotype Caller, and GATK Unified Genotyper were applied to call single nucleotide variations (SNVs) and indels, which were mapped to the genome location using the UCSC Genome Browser (http://genome.ucsc.edu). The Refseq database (Human Reference Genome version hg19) was used as the reference genome. The filtration of detected SNVs and indels was performed by homemade pipeline with the above software. Details for DNA sequencing are provided in the Supporting Information Methods.

### 2.3 RNA sequencing

Total RNA was extracted from frozen tumor tissue by Trizol and RNeasy Mini Kit (Qiagen). RNA quantity was assessed on Nanodrop, and the integrity of total RNA was estimated by RNA 6000 Nano Kit on Aligent 2100 Bioanalyzer. Among the 670 patients with DNA sequencing data, RNA sequencing was performed on qualified frozen tumor tissue from 349 patients. Read pairs were aligned to Refseq hg19 by Burrows‐Wheeler Aligner version 0.7.13‐r1126. The HTSeq was applied to generate transcript counts table files.^17^ Visual inspection was used to exclude potential false positive results. Bioinformatic analyses were performed by r 3.5.1, using R package “sva” to remove batch effect. Raw reads were normalized, and differentially expressed genes were obtained with R package “limma” (v3·38·3). Details for RNA sequencing are provided in the Supporting Information Methods.

### 2.4 Molecular classification

DLBCL genotypes were identified using the LymphGen probabilistic classification tool (R code version, https://doi.org/10.5281/zenodo.3700087) as described by Wright et al.^18^ Genetic aberrations including mutations, copy‐number alterations, and fusions were analyzed and integrated. The probabilities of each GenClass‐defined genotype were calculated in 325 patients with WES/WGS data. Identified patients were assigned into six genotypes (MCD, BN2, N1, EZB, ST2, and A53) according to the classification tool.

### 2.5 GSEA

GSEA results were presented as the upregulation or downregulation of the desired gene set using GSEA v4.0.1 software and Molecular Signature Database.^19^, ^20^ The metric method used to rank the genes was Signal2Noise by default. In this article, the phenotype which contained at least seven samples was labeled as the permutation type. Analysis was run on 1000 permutations to assess the statistical significance of the enrichment score, as recommended by the GSEA team (http://www.broadinstitute.org/gsea). Pathways were considered statistically significant when the *P* value was <.05, and the false discovery rate was <.25.

### 2.6 TIP

The activity score of anti‐tumor immunity was generated using tracking tumor immunophenotyping (TIP, http://biocc.hrbmu.edu.cn/TIP ^21^) method, which contains 178 signature genes and 23 signature gene sets involved in the cancer‐immunity cycle as described in published studies and allows the discrimination of the recruitment of specific T‐cell subsets.^22^ With the gene expression data, the activity scores of the gene sets were calculated separately, based on their stimulatory or inhibitory role in the anti‐tumor immune response. The final score of each signature gene set for each individual sample was calculated by examining the difference between the normalized scores of stimulatory gene set and inhibitory gene set.

### 2.7 Zebrafish models

Zebrafish were housed in an aquatic system equipped with continuously aeration and filtration. Wild‐type Tuebingen zebrafish were used in this study. The embryos were incubated at 28.5°C. At 48‐hour postfertilization, 500‐1000 Dil‐labeled primary DLBCL cells were carefully injected into the perivitelline space of each larva after anesthetized. Primary DLBCL cells with *MYD88* mutations or *B2M* mutations were injected into zebrafish as experimental groups, while those with *MYD88* wildtype and *B2M* wildtype were used as the control group. The injected xenografts were immediately transferred to an incubator and maintained at 34°C. At 24‐hour postinjection, successful injected xenografts were confirmed by immunofluorescence: nine xenografts in control group, 21 xenografts in *MYD88* mutation group, and 23 xenografts in *B2M* mutation group. Zebrafish of control and experimental groups were maintained separately until the end of experiment. At 6 dpi, the number of xenografts with lymphoma invasion of the particular organs away from the injection sites was analyzed and recorded. Human tissue processing was performed as described by Fior et al.^23^ The tissue used for zebrafish patient‐derived tumor xenograft model establishment was obtained from Shanghai Ruijin Hospital with written informed consent. All animal experiments were approved by the Animal Care and Use Committee of Shanghai Jiao Tong University and conducted in conformity with the rules of the Committee on Animal Care of Shanghai, China.

### 2.8 Immunohistochemistry and immunofluorescence

Immunohistochemistry of patient tumor tissue was performed on 5 μm paraffin sections using antibody against HLA‐A (abcam, ab52922, 1:200), HLA‐B (abcam, ab225636, 1:200), HLA‐C (abcam, ab193432, 1:200), and CXCR4 (abcam, ab181020, 1:200). Immunohistochemistry of zebrafish model tissues was performed on 3 μm agarose sections,^23^ stained with H&E or primary antibodies of anti‐human CD19 (abcam, ab134114, 1:200) and anti‐human CXCR4 (abcam, ab1670, 1:150). Protein expression levels were recorded according to percentage of stained cells. DLBCL exhibiting a >50% reduction of HLA expression (evidently reduced relative to surrounding non‐malignant cells) was categorized as reduced HLA expression, otherwise it was classified as positive expression, as previously reported.^24^ CXCR4 staining ≥20% was referred as positive.

For immunofluorescence, zebrafish model tissues were fixed in 4% formaldehyde and stored in methanol at −20°C. Tumor cells were labeled by Dil, and nuclei were counterstained with DAPI. Pictures were captured in a Zeiss880 microscope.

### 2.9 Statistical analysis

The best cutoffs of continuous variables were obtained by maximizing the Youden's index using receiver operating characteristic (ROC) curve analysis. Baseline characteristics of patients were ascertained using Pearson's *χ* ^2^ test or Fisher's exact test. Differences of immunity activity scores and normalized gene expression in two groups were analyzed using Mann‐Whitney *U* test. Progression‐free survival (PFS) was measured from the date of diagnosis to the date when disease progression/relapse was recognized or the date of last follow‐up. Overall survival (OS) was calculated from the date of diagnosis to the date of death or the date of last follow‐up. Survival functions were generated with the Kaplan‐Meier method and compared by the log‐rank test. Univariate hazard was estimated using the Cox regression method. Significant variables on univariate analysis were kept in multivariate set. Statistical significance was defined as *P* < .05. All *P* values in this manuscript were reported without mathematical correction. The above statistical analyses were performed by Statistical Package for the Social Sciences (SPSS) 26.0 software (SPSS Inc., Chicago, IL).

## 3 RESULTS

### 3.1 Clinical and prognostic significance of DLBCL patients with multiple ENI

A total of 1960 patients with newly diagnosed DLBCL were analyzed, including 662 patients without ENI, 902 patients with single ENI, and 396 patients with multiple ENI. The clinical characteristics of the patients are summarized in Table 1. Multiple ENI was significantly associated with poor performance status (*P* < .0001), advanced Ann Arbor stage (*P* < .0001), elevated serum LDH (*P* < .0001), as well as increased prevalence of the non‐GCB subtype (*P* = .0059) compared to those without ENI and with single ENI. No significant differences in Epstein‐Barr virus‐encoded RNA positivity (7.0% or 13/186 vs 8.4% or 50/598; *P* = .5478) or the proportion of double/triple‐hit lymphomas indicating BCL2/BCL6/MYC rearrangements (2.6% or 3/114 vs 1.7% or 6/360; *P* = .4546) were observed between patients with multiple ENI and those without ENI and with single ENI. Meanwhile, patients with multiple ENI showed a remarkable decrease in overall response rate (75.5%) and 2‐year OS rate (61.8%) relative to those without ENI (89.3% and 81.3%, respectively) and with single ENI (84.6% and 80.4%, respectively; both *P* < .0001) following R‐CHOP treatment.

**TABLE 1:** Clinical characteristics of the patients with DLBCL (n = 1960)

Characteristics	Number of extranodal involvement			*P* value^a^	*P* value^b^	*P* value^c^
	None (n = 662)	Single (n = 902)	Multiple (n = 396)			
Gender				.3320	.0164	.0520
Male	371 (56.0%)	468 (51.9%)	234 (59.1%)			
Female	291 (44.0%)	434 (48.1%)	162 (40.9%)			
Age				.1670	.296	.1910
≤60 year	372 (56.2%)	493 (54.7%)	204 (51.5%)			
>60 year	290 (43.8%)	409 (45.3%)	192 (48.5%)			
ECOG score				<.0001	.0119	<.0001
0‐1	629 (95.0%)	792 (87.8%)	327 (82.6%)			
≥2	33 (5.0%)	110 (12.2%)	69 (17.4%)			
Ann Arbor stage				<.0001	<.0001	<.0001
I‐II	443 (66.9%)	503 (55.8%)	59 (14.9%)			
III‐IV	219 (33.1%)	399 (44.2%)	337 (85.1%)			
LDH				<.0001	<.0001	<.0001
Normal	372 (56.2%)	536 (59.4%)	113 (28.5%)			
Elevated	290 (43.8%)	366 (40.6%)	283 (71.5%)			
Cell of origin (Hans)				.0181	.0087	.0059
GCB	210/546 (38.5%)	280/720 (38.9%)	107/349 (30.7%)			
Non‐GCB	336/546 (61.5%)	440/720 (61.1%)	242/349 (69.3%)			
EBV‐encoded RNA				.9652	.3085	.5478
Positive	19/276 (6.9%)	31/322 (9.6%)	13/186 (7.0%)			
Negative	257/276 (93.1%)	291/322 (90.4%)	173/186 (93.0%)			
Double‐hit/triple‐hit				.6530	.6958	.4546
Yes	2/156 (1.3%)	4/204 (2.0%)	3/114 (2.6%)			
No	154/156 (98.7%)	200/204 (98.0%)	111/114 (97.4%)			
Overall response rate	491/550 (89.3%)	582/688 (84.6%)	234/310 (75.5%)	<.0001	.0006	<.0001
2‐year OS rate	81.3%	80.4%	61.8%	<.0001	<.0001	<.0001

^a^ *P* value indicated difference between lymphoma with multiple extranodal involvement and lymphoma without extranodal involvement.

^b^ *P* value indicated difference between lymphoma with multiple extranodal involvement and lymphoma with single extranodal involvement.

^c^ *P* value indicated difference between lymphoma with multiple extranodal involvement and lymphoma without/with single extranodal involvement.

Abbreviations: ECOG, Eastern Cooperative Oncology Group; GCB, germinal center B‐cell; LDH, lactate dehydrogenase; OS, overall survival.

**FIGURE 2:**
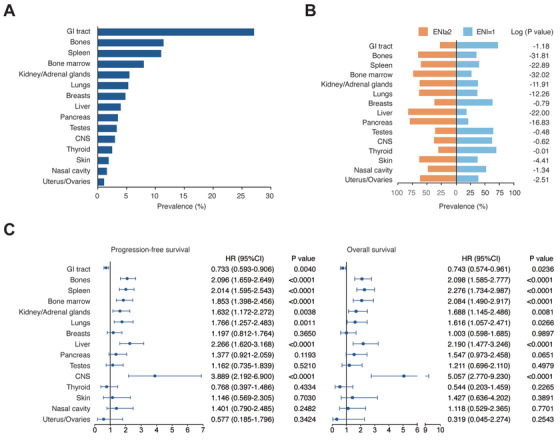
Distribution and clinical outcomes according to sites of extranodal invasion in DLBCL. A, Distribution according to sites of extranodal invasion (GI tract, bones, spleen, bone marrow, kidney/adrenal glands, lungs, breasts, liver, pancreas, testes, CNS, thyroid, skin, nasal cavity, and uterus/ovaries in DLBCL patients (n = 1960). B, Prevalence of multiple ENI and single ENI according to sites of extranodal invasion. *P* values comparing different prevalence in two groups are indicated at the right. C, Univariate analysis of predictors for PFS and OS according to sites of extranodal invasion in DLBCL patients (n = 1701). Hazard ratios (HR), 95% confidence intervals (95% CI), and *P* values are indicated on the right of each forest plot

According to specific extranodal organs, DLBCL was observed most often in the GI tract (27.1%), followed by the bones (11.4%), spleen (11.0%), bone marrow (8.0%), kidney/adrenal glands (5.5%), lungs (5.3%), breasts (4.8%), liver (4.0%), pancreas (3.5%), testes (3.3%), CNS (3.0%), thyroid (2.5%), skin (1.9%), nasal cavity (1.6%), and uterus/ovaries (1.1%; Figure 2A). Multiple ENI was more frequently observed in extranodal organs, including the bones, spleen, bone marrow, kidney/adrenal glands, lungs, liver, pancreas, skin, nasal cavity, and uterus/ovaries compared to single ENI (Figure 2B). No significant difference was found between the extranodal invasion determined by PET‐CT and by CT/MRI (Table S1).

Among the 1701 patients treated with R‐CHOP, the median follow‐up time was 30.7 months (0.1‐203.9 months). Using univariate analysis, lymphoma invasion of the bones, spleen, bone marrow, kidney/adrenal glands, lungs, liver, and CNS were unfavorable predictors of both PFS and OS, while lymphoma invasion of the GI tract was related to favorable outcomes (Figure 2C). Significant factors from the univariate selection were included in the multivariate analysis to identify the best predictor set. ENI of the bones, spleen, bone marrow, liver, and CNS (PFS: hazard ratio [HR]  =  1.573, 95% CI 1.207‐1.797, *P* = .0001; OS: HR  =  1.432, 95% CI 1.125‐1.823, *P* = .0035) were independent prognostic factors of inferior PFS and OS when adjusted by standard prognostic factors, including age (PFS: HR  =  1.212, 95% CI 1.030‐1.474, *P* = .0222; OS: HR  =  1.577, 95% CI 1.264‐1.967, *P* < .0001), performance status (PFS: HR  =  1.776, 95% CI 1.402‐2.250, *P* < .0001; OS: HR  =  1.976, 95% CI 1.506‐2.594, *P* < .0001), Ann Arbor stage (PFS: HR  =  1.825, 95% CI 1.467‐2.272, *P* < .0001; OS: HR  =  1.789, 95% CI 1.357‐2.360, *P* < .0001), and serum LDH (PFS: HR  =  1.876, 95% CI 1.535‐2.292, *P* < .0001; OS: HR  =  2.498, 95% CI 1.929‐3.233, *P* < .0001; Table 2).

**TABLE 2:** Multivariate analysis for OS and PFS (n = 1701)

	PFS		OS	
	*P* value	HR (95% CI)	*P* value	HR (95% CI)
Age, >60 year	.0222	1.212 (1.030‐1.474)	<.0001	1.577 (1.264‐1.967)
ECOG PS, ≥2	<.0001	1.776 (1.402‐2.250)	<.0001	1.976 (1.506‐2.594)
Ann Arbor stage, III‐IV	<.0001	1.825 (1.467‐2.272)	<.0001	1.789 (1.357‐2.360)
Serum LDH	<.0001	1.876 (1.535‐2.292)	<.0001	2.498 (1.929‐3.233)
Specific extranodal disease*	.0001	1.573 (1.207‐1.797)	.0035	1.432 (1.125‐1.823)

* Disease in bone, spleen, bone marrow, liver, or central nervous system.

Abbreviations: HR, hazard ratio; OS, overall survival; PFS, progression‐free survival.

### 3.2 Oncogenic mutations and signaling pathway alterations related to multiple ENI

**FIGURE 3:**
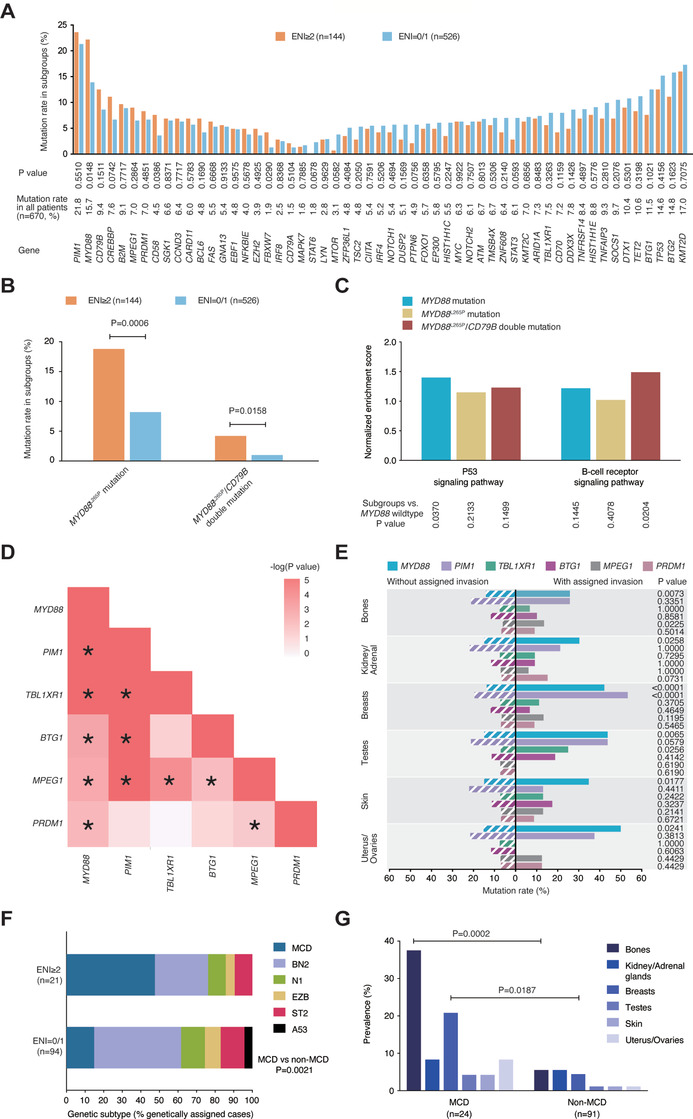
Relationship between oncogenic mutations and multiple ENI in DLBCL. A, Prevalence of genetic mutations in patients with multiple ENI (n = 144) and without ENI/with single ENI (n = 526) of DLBCL. Lower graph indicates genes, mutation rates in all patients, as well as *P* values comparing different prevalence in two groups. B, Prevalence of *MYD88* ^L265P^ mutations alone or with *CD79B* mutations in patients with multiple ENI (n = 144) and without ENI/with single ENI (n = 526). *P* values comparing different prevalence in two groups are indicated above the columns. C, Pathways and normalized enrichment scores for patients assigned to *MYD88* mutations, *MYD88* ^L265P^ mutations alone, or with *CD79B* mutations. Lower graph shows *P* values of dysregulated pathways in each group, as compared to *MYD88* wildtype group. D, Correlations of *MYD88* mutations and co‐occurring mutations visualized in a correlation matrix. Bivariate correlation was calculated using Spearman's rank correlation. Asterisks mark all bivariate correlations that are significant (*P* < .05). E, Prevalence of *MYD88* mutations and co‐occurring mutations in extranodal invasion of bones, kidney/adrenal glands, breasts, testes, skin, and uterus/ovaries. *P* values comparing mutation rates in groups with or without assigned invasion are indicated on the right. F, Prevalence of DLBCL subtypes classified by LymphGen in patients with multiple ENI (n = 21) and without ENI/with single ENI (n = 94). G, Extranodal involvement in MCD subtype (n = 24) and non‐MCD subtypes (n = 91). *P* values comparing different percentages are indicated in subgroups

Oncogenic mutations closely related to DLBCL were analyzed in 670 patients, including 216 cases by WES, 109 cases by WGS, and 345 cases by targeted sequencing. As shown in Figure 3A, *MYD88* mutations were significantly increased in patients with multiple ENI (22.2% or 32/144) compared to those without ENI and with single ENI (13.9% or 73/526; *P* = .0148). The *MYD88* ^L265P^ mutation, either alone (18.8% or 27/144) or with the *CD79B* mutation (4.2% or 6/144), was more frequently observed in patients with multiple ENI than those without ENI and with single ENI (8.2% or 43/526; *P* = .0006 and 1.0% or 5/526; *P* = .0158, respectively). As revealed by GSEA (Figure 3C and Figure S1), P53 signaling pathway was upregulated based on *MYD88* mutations (*P* = .0370), and B‐cell receptor signaling pathway was upregulated according to *MYD88* ^L265P^ */CD79B* double mutation (*P* = .0204).

*MYD88* mutations also co‐occurred with mutations in *PIM1*, *TBL1XR1*, *BTG1*, *MPEG1*, and *PRDM1*. Among the 105 patients with *MYD88* mutations, 53 patients (50.5%) had a *PIM1* mutation, 22 patients (21.0%) had a *TBL1XR1* mutation, 22 patients (21.0%) had a *BTG1* mutation, 15 patients (14.3%) had a *MPEG1* mutation, and 14 patients (13.3%) had a *PRDM1* mutation. Correlations between MYD88‐related genes included *PIM1* and *BTG1* (34 cases), *PIM1* and *TBL1XR1* (28 cases), *PIM1* and *MPEG1* (24 cases), *TBL1XR1* and *MPEG1* (11 cases), *BTG1* and *MPEG1* (11 cases), and *MPEG1* and *PRDM1* (seven cases, Figure 3D). Interestingly, *MYD88* mutations were significantly increased in patients with lymphoma invasion of the bones (25.8% or 23/89; *P* = .0073), kidney/adrenal glands (30.3% or 10/33; *P* = .0258), breasts (42.2% or 19/45; *P* < .0001), testes (43.8% or 7/16; *P* = .0065), skin (34.8% or 8/23; *P* = .0177), and uterus/ovaries (50.0% or 4/8; *P* = .0241) compared to those without invasion of the bones (14.1% or 82/581), kidney/adrenal glands (14.9% or 95/637), breasts (13.8% or 86/625), testes (15.0% or 98/654), skin (15.0% or 97/647), and uterus/ovaries (15.3% or 101/662). *PIM1* mutations were higher in patients with breast invasion than those without invasion (53.3% or 24/45 vs 19.5% or 122/625; *P* < .0001). *TBL1XR1* mutations were higher in patients with testes invasion than those without invasion (25.0% or 4/16 vs 7.0% or 46/654; *P* = .0256). *MPEG1* mutations were higher in patients with bone invasion than those without invasion (13.5% or 12/89 vs 6.0% or 35/581; *P* = .0225).

Genetic aberrations related to other prognosis‐related extranodal organs are shown in Figure S2. Increased *TET2* mutations (15.4% vs 8.9%; *P* = .0292) but decreased mutations in *DUSP2* (1.8% vs 6.2%; *P* = .0245), *MPEG1* (3.0% vs 8.5%; *P* = .0146), and *HIST1H1E* (4.7% vs 10.1%; *P* = .0287), *MYD88* (7.7% vs 18.3%; *P* = .0009), and *PIM1* (13.6% vs 24.5%; *P* = .0025) were observed in patients with GI tract invasion compared to those without invasion (Figure S2A). Mutations in *KMT2D* (25.6% vs 16.0%; *P* = .0371), *SOCS1* (16.7% vs 8.8%; *P* = .0394), *BCL6* (10.3% vs 4.0%; *P* = .0240), and *IRF8* (7.7% vs 1.9%; *P* = .0089) were higher in patients with spleen invasion than those without invasion (Figure S2B). Mutations in *FAS* (12.8% vs 5.0%; *P* = .0343) and *NFKBIE* (10.6% vs 3.5%; *P* = .0315) were higher in patients with lung invasion than those without invasion (Figure S2C). Mutations in *BCL6* (21.7% vs 4.2%; *P* = .0033) were higher in patients with liver invasion than those without invasion (Figure S2D).

One hundred twenty‐three (37.9%) of the 325 patients were genetically classified (Figure S2E). MCD genotype was more frequently observed in patients with multiple ENI than those without ENI and with single ENI (47.6% or 10/21 vs 14.9% or 14/94; *P* = .0021; Figure 3F). Similarly, invasion of the bones (37.5% vs 5.5%), kidney/adrenal glands (8.3% vs 5.5%), breasts (20.8% vs 4.4%), testes (4.2% vs 1.1%), skin (4.2% vs 1.1%), and uterus/ovaries (8.3% vs 1.1%) was relatively increased in MCD genotype, as compared to other DLBCL genotypes. MCD genotype was closely related to invasion of the bones and breasts (*P* = .0002 and *P* = .0187, respectively; Figure 3G). None of the DLBCL genotypes was significantly associated with invasion of the GI tract, spleen, lungs, or liver (Figure S2F).

### 3.3 Tumor microenvironment alterations related to multiple ENI

**FIGURE 4:**
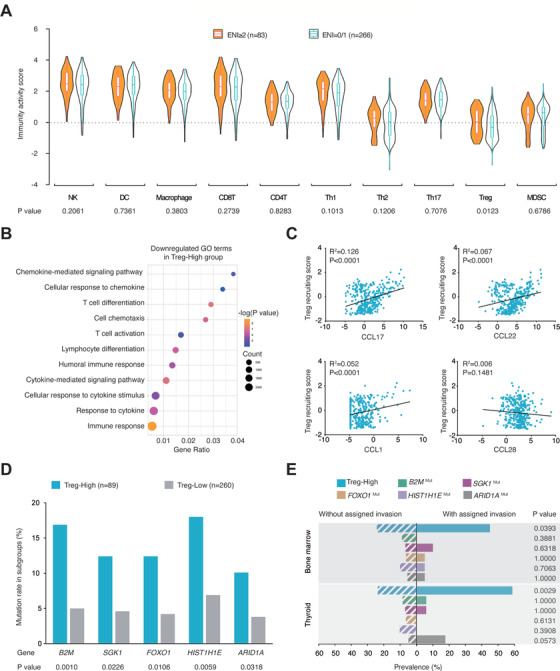
Relationship between intratumor immune cells and multiple ENI in DLBCL. A, Immunity activity scores of indicated immune cells in patients with multiple ENI (n = 83) and without ENI/with single ENI (n = 266). Lower graph indicates *P* values comparing different scores in two groups. B, Downregulated gene ontology (GO) terms in Treg‐high group (n = 89), as compared to Treg‐low group (n = 260). Color of points indicates −log (*P* value) of dysregulated pathways in two groups. Size of points indicates number of genes included in each gene set. C, Correlations between the chemokines corresponding to Treg cell recruitment and Treg‐recruiting score. *P* values and *R* ^2^ values are indicated in each plot. D, Mutation rates of *B2M*, *SGK1*, *FOXO1*, *HIST1H1E*, and *ARID1A* in Treg‐high group and Treg‐low group. Lower graph indicates *P* values comparing mutation rates in two groups. E, Prevalence of Treg‐high subgroup and gene mutations in extranodal invasion of the bone marrow and thyroid. *P* values comparing prevalence in groups with or without assigned invasion are indicated on the right

To further determine the role of immune cells in ENI, the activity scores of tumor immune cell recruitment were revealed by TIP method using RNA sequencing data from 349 patients, including 83 patients with multiple ENI and 266 patients without ENI and with single ENI (Figure 4A). Multiple ENI was significantly associated with increased Treg cell recruiting activity (*P* = .0123), while other immune cell subsets showed no significant differences, including natural killer cells, dendritic cells, macrophages, CD8+ T cells, CD4+ T cells, Th1 cells, Th2 cells, Th17 cells, and MDSCs. All patients were subsequently divided into two groups, Treg‐high (n = 89) and Treg‐low (n = 260), according to the optimal cutoff estimated by ROC curve analysis. Gene ontology analysis revealed that T‐cell immunity (T‐cell differentiation, T‐cell activation, and lymphocyte differentiation), chemokine signaling (chemokine‐mediated signaling, cellular response to chemokine, and cell chemotaxis), and other immune‐associated signaling (cytokine‐mediated signaling pathway, cellular response to cytokine stimulus, response to cytokine, and immune response) were downregulated in Treg‐high patients compared to Treg‐low patients (Figure 4B). The relationship between chemokine expression with Treg cell recruitment and Treg recruiting score was analyzed. Among the main chemokines, CCL17, CCL22, and CCL1 showed positive linear correlations with Treg‐recruiting score (all *P* < .0001; Figure 4C). The genes that were more frequently mutated in the Treg‐high group than in the Treg‐low group included *B2M* (16.9% vs 5.4%; *P* = .0010), *SGK1* (12.4% vs 5.0%; *P* = .0226), *FOXO1* (12.4% vs 4.2%; *P* = .0106), *HIST1H1E* (18.0% vs 6.9%; *P* = 0.0059), and *ARID1A* (10.1% vs 3.8%; *P* = .0318; Figure 4D). The proportion of Treg‐high patients was significantly associated with lymphoma invasion of the bone marrow (45.0% or 9/20) and thyroid (58.8% or 10/17) compared to those without invasion of the bone marrow (24.2% or 80/329; *P* = .0393) and thyroid (23.7% or 79/332; *P* = .0029; Figure 4E). The tumor microenvironment status related to other prognosis‐related extranodal organs was also assessed. Increased neutrophils but decreased Th1 and dendritic cells were observed in patients with GI tract invasion relative to those without invasion (Figure S2E).

**FIGURE 5:**
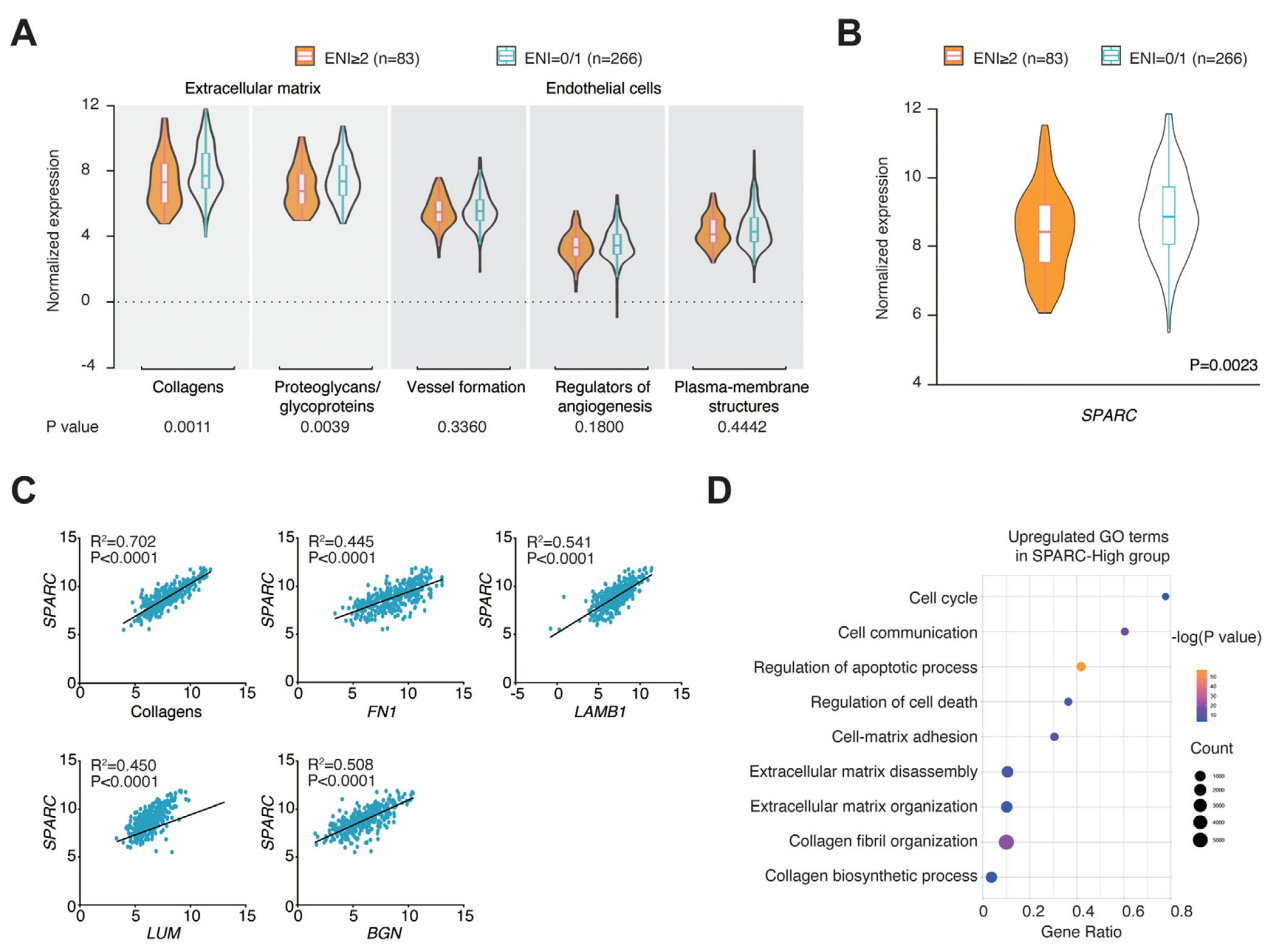
Relationship between extracellular matrix (ECM) and multiple ENI in DLBCL. A, Normalized expression of genes encoding ECM and endothelial cells in patients with multiple ENI (n = 83) and without ENI/with single ENI (n = 266). Lower graph indicates *P* values comparing different scores in two groups. B, *SPARC* expression in patients with multiple ENI (n = 83) and without ENI/with single ENI (n = 266). C, Correlations between other ECM components and *SPARC* expression. *P* values and *R* ^2^ values are indicated in each plot. D, Upregulated gene ontology (GO) terms in SPARC‐high group (n = 157), as compared to SPARC‐high group (n = 192). Color of points indicates –log (*P* value) of dysregulated pathways in two groups. Size of points indicates number of genes included in each gene set

To further investigate the role of ECM and endothelial cells on ENI, the expression of genes encoding ECM molecules, including collagens (*COL1A1*, *COL1A2*, *COL3A1*, *COL4A1*, *COL5A2*, and *COL6A3*) and proteoglycans/glycoproteins (*LUM*, *BGN*, *LAMB1*, *FN1*, and secreted protein acidic and rich in cysteine [*SPARC*]), as well as genes encoding endothelial cells (*ADGRF5*, *CAV1*, *CAV2*, *EGFL7*, *EHD2*, *ERG*, *GRB10*, *ITGA9*, *KDR*, *MMRN2*, *PECAM1*, *ROBO3*, *SPARCL1*, *TEK*, and *VWF*), were screened using RNA sequencing data. Patients with multiple ENI presented decreased expression levels of genes encoding collagens and proteoglycans/glycoproteins compared to those without ENI and with single ENI (*P* = .0011 and *P* = .0039, respectively; Figure 5A). SPARC, a key component of the ECM, displayed significantly lower expression in patients with multiple ENI than those without ENI and with single ENI (*P* = .0023; Figure 5B), which correlated with decreased levels of the above ECM components, including collagens, lumican, byglican, laminin, and fibronectin (all *P* < .0001; Figure 5C). All patients were subsequently divided into two groups: SPARC‐low (n = 157) and SPARC‐high (n = 192), according to the optimal cutoff estimated by ROC curve analysis. Gene ontology analysis revealed that cell death (cell cycle, regulation of apoptotic process, and regulation of cell death), ECM formation (ECM disassembly, ECM organization, collagen fibril organization, and collagen biosynthetic process), and cell‐stroma interaction (cell communication and cell‐matrix adhesion) were upregulated in SPARC‐high patients compared to SPARC‐low patients (Figure 5D).

### 3.4 MHC class I expression related to multiple ENI

The influences of ENI‐related mutations on MHC class I expression were evaluated. Decreased *HLA‐A* expression by RNA sequencing and reduced positivity of HLA‐A expression by immunohistochemistry were significantly associated with mutations in *SGK1* (*P* = .0244 and *P* = .0061, respectively) and *HIST1H1E* (*P* = .0201 and *P* = .0248, respectively; Figure S3A). Similarly, decreased *HLA‐B* expression and reduced positivity of HLA‐B expression were significantly associated with mutations in *MYD88* (*P* = .0056 and *P* = .0093, respectively), *PIM1* (*P* = .0030 and *P* = .0015, respectively), and *TBL1XR1* (*P* = .0068 and *P* = .0106, respectively; Figure S3B). Decreased *HLA‐C* expression and reduced positivity of HLA‐C expression were significantly associated with mutations in *FOXO1* (*P* = .0134 and *P* = .0038, respectively) and *ARID1A* (*P* = .0204 and *P* = .0204, respectively; Figure S3C).

### 3.5 Chemokine receptor expression related to organotropic lymphoma invasion

**FIGURE 6:**
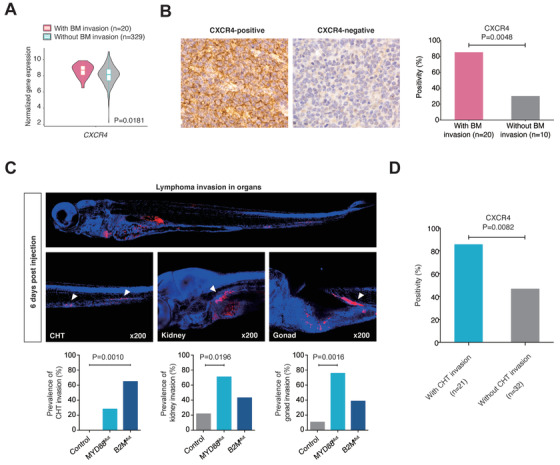
Relationship between chemokine receptor expression and organotropic lymphoma invasion. A, Normalized gene expression of *CXCR4* in patients with or without bone marrow (BM) invasion. *P* values comparing different gene expression are indicated in subgroups. B, Immunohistochemical images and positivity of CXCR4 expression in patients with or without bone marrow (BM) invasion on patient tumor samples. C, Lymphoma invasion to caudal hematopoietic tissue (CHT), kidney, and gonad of zebrafish models. Immunofluorescence images are shown for organ recognition. Arrowheads indicate corresponding structures. *P* values comparing different percentages are indicated in subgroups. D, Positivity of CXCR4 expression in zebrafish with or without CHT invasion. CXCR4 expression was evaluated by Immunohistochemistry (x400 magnification). *P* values comparing different percentages are indicated in subgroups

The correlation between chemokine receptor expression and ENI of prognosis‐related organs was further analyzed. Based on RNA sequencing data, increased *CXCR4* expression was associated with bone marrow invasion (*P* = .0181; Figure 6A). Using immunohistochemistry, increased positivity of CXCR4 was observed in patients with bone marrow invasion than those without invasion (85.0% or 17/20 vs 30.0% or 3/10; *P* = .0048; Figure 6B).

In zebrafish injected with primary DLBCL cells, lymphoma invasion of the kidneys, gonads, and caudal hematopoietic tissue (CHT) was assessed by immunofluorescence. Representative images of lymphoma invasion in organs are shown in Figure 6C. A significantly increased percentage of CHT invasion was observed in zebrafish models with *B2M* mutations compared to those without mutations (65.2% or 15/23 vs 0.0% or 0/9; *P* = .0010). Increased percentages of kidney invasion (71.4% or 15/21 vs 22.2% or 2/9; *P* = .0196) and gonad invasion (76.2% or 16/21 vs 22.2% or 2/9; *P* = .0016) were observed in zebrafish models with *MYD88* mutations compared to those without mutations (Figure 6C). With respect to organ‐specific dissemination, the immunohistochemistry study showed an increased percentage of positive CXCR4 expression in zebrafish models with CHT invasion compared to those without CHT invasion (85.7% or 18/21 vs 46.9% or 15/32; *P* = .0082; Figure 6D).

## 4 DISCUSSION

Multiple ENI is closely related to high tumor burden that manifests from poor performance status, advanced disease stage, and elevated serum LDH, leading to poor response to rituximab‐containing immunochemotherapy and adverse clinical outcomes in DLBCL. Our data displayed an increased prevalence of GI tract involvement as compared to the previous report.^4^ This observation could potentially be related to the improved sensitivity of CT scanning techniques and the increasing application of PET‐CT scanning, both of which might enhance the efficiency for the determination of certain abnormalities including GI tract involvement.^25^ Correspondingly, a more recent study revealed the prevalence of gastric involvement at 12.8% and intestinal involvement at 15.5% of the patients, respectively.^26^ A multicenter study in China also indicated that 25.2% of the patients have GI tract involvement, which is consistent with our findings.^27^

Based on a large patient cohort and genomic data, we revealed the divergent survival status and genetic alterations for DLBCL in terms of specific extranodal organs. Clinically, in addition to the bone marrow, CNS, liver, and lungs defined by NCCN‐IPI,^6^ lymphoma invasion of the bones, spleen, and kidney/adrenal glands were unfavorable prognostic factors. This observation was consistent with another large cohort of 1221 DLBCL patients showing that bone, spleen, kidney, and adrenal gland involvement, whether primary or secondary, were associated with advanced stage and inferior prognosis.^3^ Conversely, as the most common extranodal site, GI tract involvement indicated favorable prognosis, which remains controversial among previous studies.^3^, ^28^, ^29^ As for genetic alterations, we provided genomic evidence corresponding to clinical data in extranodal DLBCL. Oncogenic mutations were frequently observed in patients with lymphoma invasion of the bones, kidney/adrenal glands (*MYD88*),^30^ spleen (*BCL6*, *IRF8*, *KMT2D*, and *SOCS1*),^31^, ^32^, ^33^, ^34^ lungs (*FAS* and *NFKBIE*),^35^, ^36^ and liver (*BCL6*),^31^ but less often in patients with GI invasion (*MYD88, PIM1*, *MEPG1, DUSP2*, and *HIST1H1E*).^8^, ^30^, ^37^, ^38^ Therefore, the biological behavior of lymphoma cells may contribute to ENI in DLBCL.

Among oncogenic mutations, *MYD88* is implicated in tumorigenesis through proinflammatory mechanisms^39^ and has recently been identified as a molecular subgroup of DLBCL with poor prognosis.^40^ Our results showed a relationship between *MYD88* mutations and multiple ENI, indicating another important biomarker of ENI in DLBCL. Dysregulation of the P53 and B‐cell receptor signaling pathways was present in *MYD88*‐mutated DLBCL, which resulted in constitutive activation of nuclear factor kappa‐B (NF‐κB) pathway, resistance to chemotherapy, but sensitivity to the BTK inhibitor.^41^, ^42^, ^43^ Of note, a series of gene mutations co‐occurred with *MYD88*, including *PIM1*, *TBL1XR1*, *BTG1*, *MPEG1*, and *PRDM1*, all of which presented high frequency in the MCD subtype defined by Schmitz et al, contributing to NF‐κB activation in a B cell receptor‐dependent manner.^8^ High frequencies of *PIM1* ^44^ and *TBL1XR1* ^45^ mutations were also observed in breast and testis lymphoma, respectively. These findings confirmed the experimental data showing that *MYD88* ^L265P^ alone is insufficient to drive malignant transformation in B cells and may cooperatively induce lymphoma with other genetic events.^46^ Genetically defined‐MCD subtype was closely related to multiple ENI, in particular with breasts and bones. These observations suggest a potential link of MCD genotype and immune evasion.^18^

Tumor microenvironment plays a critical role in DLBCL progression.^10^ To our knowledge, this is the first study to analyze microenvironment influence on ENI in DLBCL. Increased intratumoral Treg cells were found in DLBCL with multiple ENI, contributing to the suppression of T‐cell and cytokine‐mediated immunity and thereby resulting in immune evasion and tumor dissemination.^47^ For gene mutations related to the accumulation of Treg cells, *B2M* has recently been identified in the immune regulation of Treg cells.^48^ *SGK1*‐*FOXO1* signaling is essential for Treg cell migration.^49^ Although the roles of *H1ST1H1E* and *ARID1A* in immune cells have not been fully identified, changes mediated by epigenetic regulations may alter the function of T cells in anti‐tumor immunity.^50^, ^51^ In parallel with the signature genes for immune cell recruitment, the expression levels of CCL17, CCL22, and CCL1 were found to be correlated with Treg cell recruiting activity. Indeed, Treg cells are chemoattracted to the tumor microenvironment by chemokine gradients, including CCR8‐CCL1 and CCR4‐CCL17/CCL22.^52^ CCL1 is a potent chemoattractant in inflammatory processes that binds to its receptor, CCR8, in order to modulate Treg cell function.^53^ CCL17 and CCL22 are well established in recruiting Treg cells and favoring tumor outgrowth. More recently, therapeutic targeting for CCR4‐CCL17/CCL22 signaling has been proven effective in reducing Treg cell accumulation and increasing immune response against tumors.^54^ As for nonimmune components of the tumor microenvironment, ECM prevents tumor cell interaction and inhibits tumor invasion. Here genes encoding ECM, particularly SPARC, one of the most important ECM components associated with favorable outcomes in DLBCL,^12^ were significantly decreased in DLBCL with multiple ENI. Therefore, ECM may act as another factor for ENI in DLBCL.

MHC class I expression is critically involved in the process of immune recognition.^55^ MHC class I family mainly includes HLA‐A, HLA‐B, and HLA‐C, essential for endogenous antigen presentation of tumor cells and subsequent recognition by the immune system. Although no significant difference in HLA expression was observed between patients with multiple ENI and those without ENI and with single ENI, loss of HLA‐A, HLA‐B, and HLA‐C was associated with ENI‐related mutations, namely HLA‐A with mutations of *SGK1* and *HIST1H1E*, HLA‐B with mutations of *MYD88*, *PIM1*, and *TBL1XR1*, and HLA‐C with mutations of *FOXO1* and *ARID1A*, indicating possible role of alterations in MHC class I expression on immune surveillance by anti‐tumor T cells in DLBCL.

To further explain the organotropic dissemination of DLBCL, we analyzed the expression of chemokine receptors in the tumor microenvironment, which promote the migration of malignant B cells,^13^ and discovered a relationship between CXCR4 and bone marrow invasion. CXCR4 functions as a homing factor for malignant cells to bone marrow^56^ and is exclusively associated with increased bone marrow infiltration in DLBCL.^57^, ^58^ The CXCR4‐CXCL12 axis is linked to poor clinical outcomes in DLBCL,^59^ and a CXCR4 antagonist has already shown tumor suppressive effects on aggressive B‐cell lymphomas in vitro.^60^ Ultimately, a better understanding of the tumor microenvironment may be helpful to control the behavior of organotropic invasion in DLBCL.

## 5 CONCLUSIONS

Extranodal DLBCL is heterogeneous in clinical and molecular features, represented by variable oncogenic mutations and tumor microenvironment alterations according to specific extranodal sites. Thus, further prospective clinical studies are necessary to establish biologically driven therapeutic strategies against ENI in DLBCL.

## AUTHOR CONTRIBUTIONS

Rong Shen and Peng‐Peng Xu performed the experiments, collected and analyzed the data, and wrote the article. Nan Wang prepared biological samples and performed the experiments. Rong Shen, Peng‐Peng Xu, Nan Wang, Hong‐Mei Yi, Lei Dong, Di Fu, Jin‐Yan Huang, and Heng‐Ye Huang recruited patients, collected study data, and prepared biological samples. Hong‐Mei Yi and Lei Dong reviewed the histopathologic diagnoses. Di Fu and Jin‐Yan Huang carried out the sequencing and participated in the validation experiments. Rong Shen and Di Fu were responsible for bioinformatics investigation. Peng‐Peng Xu gave technical support. Heng‐Ye Huang was responsible for statistical review. Anne Janin, Shu Cheng, and Li Wang gathered detailed clinical information for the study. Wei‐Li Zhao conceived the study, directed and supervised research, and wrote the manuscript.

## ETHICS APPROVAL AND CONSENT TO PARTICIPATE

The study was approved by the Shanghai Ruijin Hospital Review Board. Informed consent was obtained from all patients in accordance with the Declaration of Helsinki. All tissues used for zebrafish patient‐derived tumor xenograft model establishment were obtained from Shanghai Ruijin Hospital with written informed consent. The study was approved by the Ethics Committees of Shanghai Ruijin Hospital. All experimental procedures followed the rules of the Committee on Animal Care of Shanghai, China. All animal experiments were approved by the Animal Care and Use Committee of Shanghai Jiao Tong University.

## CONFLICT OF INTEREST

The authors declare that they have no conflict of interest.

## Supporting information


**FIGURE S1** Standard GSEA graphs for the p53 signaling pathway (upper panel) and B‐cell receptor pathway (lower panel) in *MYD88* mutations (A), *MYD88* ^L265P^ mutations alone (B), or with *CD79B* mutations (C)

**FIGURE S2** Oncogenic mutations (A‐D), molecular classification (E and F) and tumor microenvironment alterations (G) related to prognosis‐related organs. *P* values comparing different percentages are indicated in subgroups

**FIGURE S3** Normalized gene expression by RNA sequencing and positivity of HLA expression by immunohistochemistry (x400 magnification) for HLA‐A (A), HLA‐B (B), and HLA‐C (C) on patient tumor samples

Supplementary information
**TABLE S1** Extranodal involvement of patients evaluated by PET‐CT and CT/MRI

Supplementary information

Supplementary information
Supplementary methods.

## Data Availability

Genomic and gene expression data have been deposited on https://www.biosino.org/node in project OEP001143.
