# Supplementary figures and images for: Influence of oncogenic mutations and tumor microenvironment alterations on extranodal invasion in diffuse large B‐cell lymphoma
Source: Clin Transl Med. 2020 Nov 24;10(7):e221. doi: 10.1002/ctm2.221 (PMC7685246; doi:10.1002/ctm2.221)

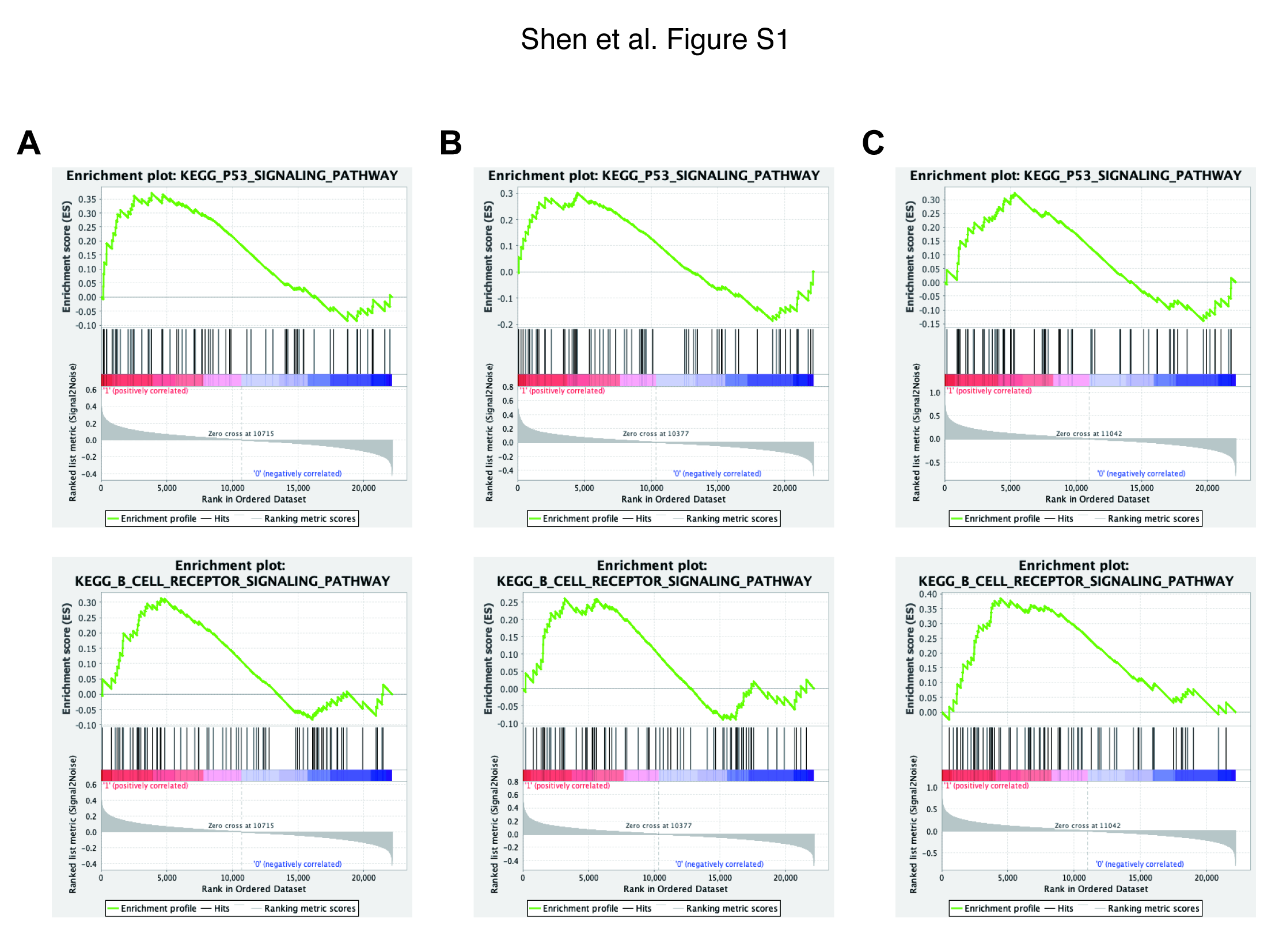

Supplement: Supplementary file 1 — FIGURE S1 Standard GSEA graphs for the p53 signaling pathway (upper panel) and B‐cell receptor pathway (lower panel) in MYD88 mutations (A), MYD88 L265P mutations alone (B), or with CD79B mutations (C) [file CTM2-10-e221-s001.tif]

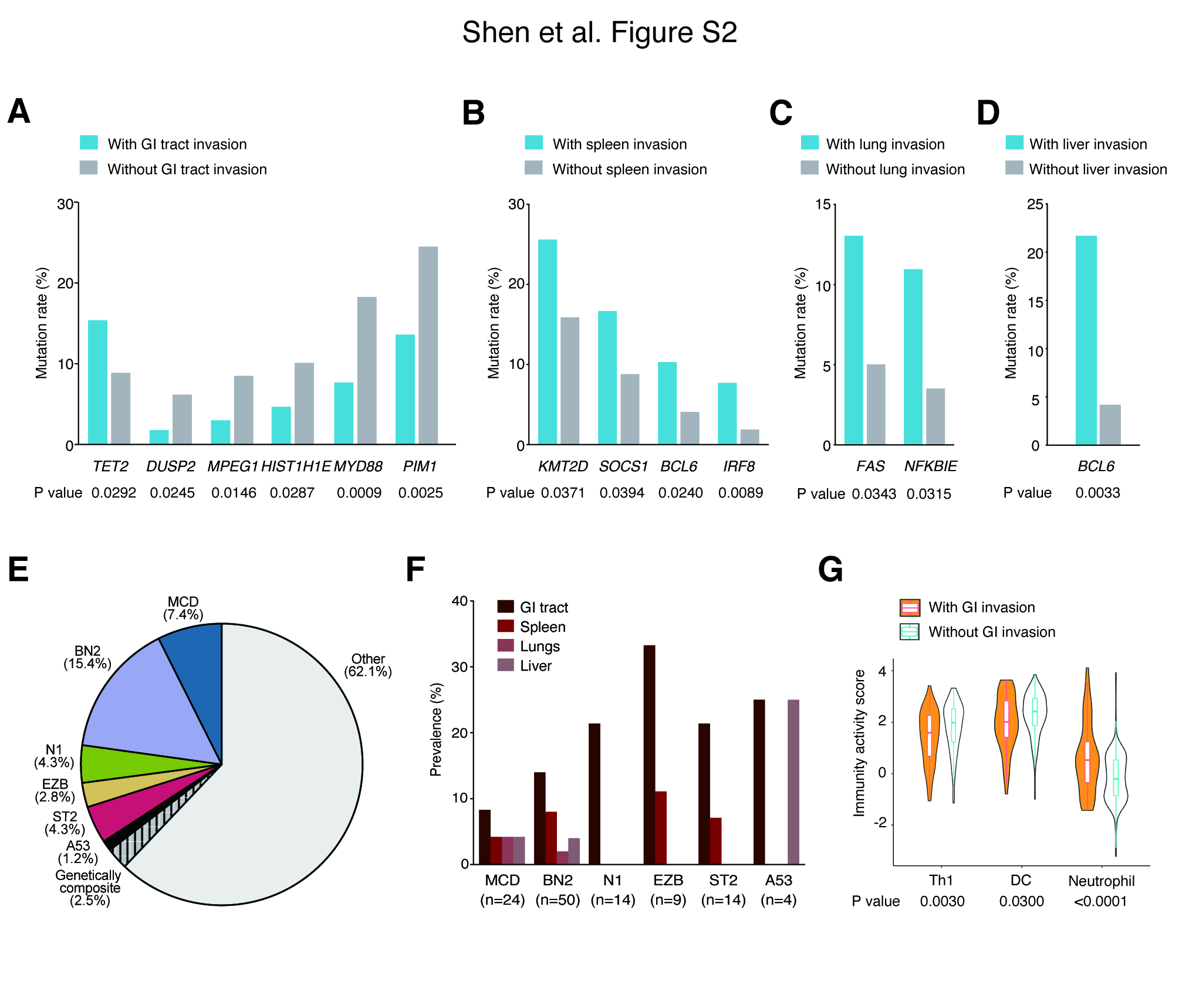

Supplement: Supplementary file 2 — FIGURE S2 Oncogenic mutations (A‐D), molecular classification (E and F) and tumor microenvironment alterations (G) related to prognosis‐related organs. P values comparing different percentages are indicated in subgroups [file CTM2-10-e221-s002.tif]

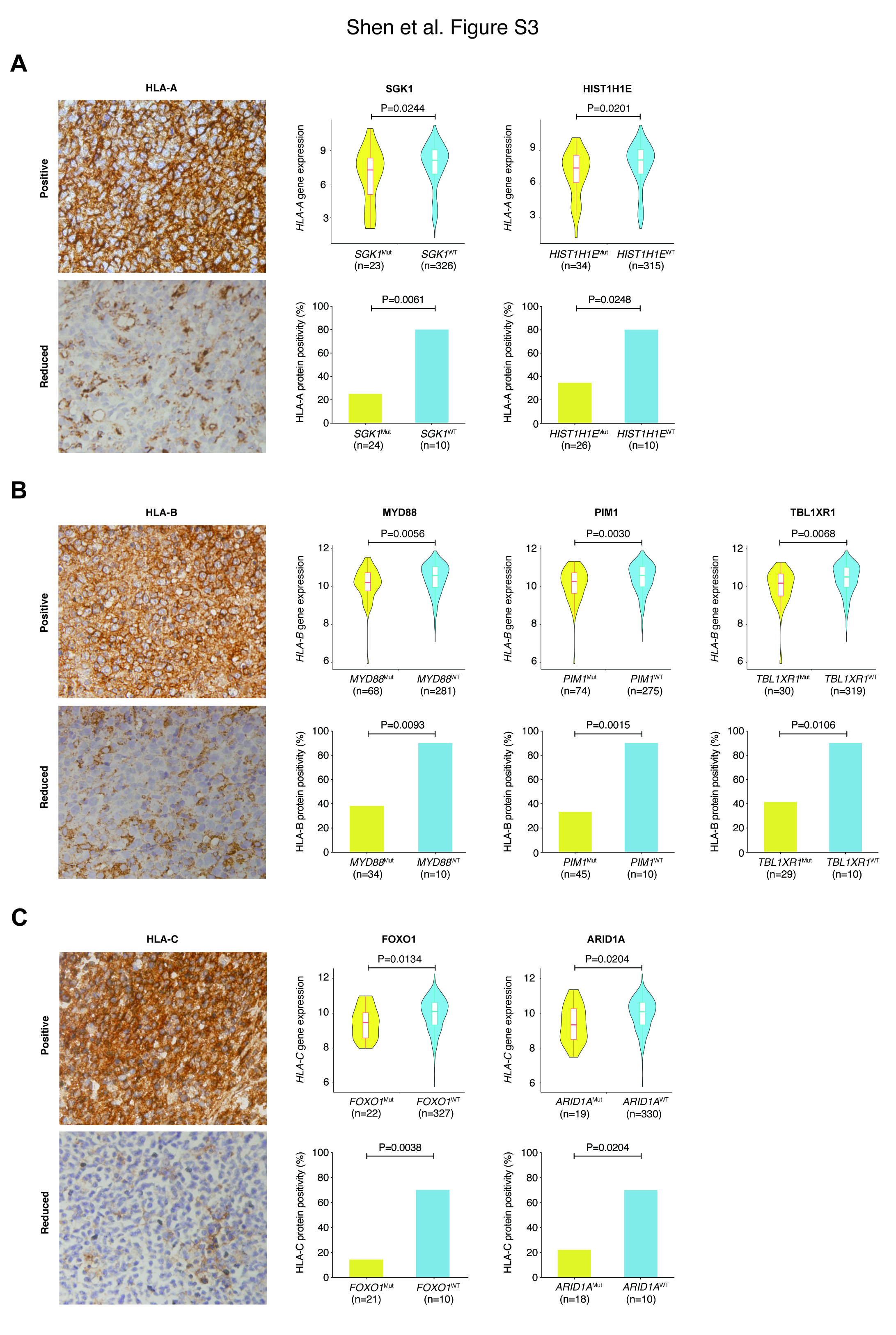

Supplement: Supplementary file 3 — FIGURE S3 Normalized gene expression by RNA sequencing and positivity of HLA expression by immunohistochemistry (x400 magnification) for HLA‐A (A), HLA‐B (B), and HLA‐C (C) on patient tumor samples [file CTM2-10-e221-s003.tif]
