# Supplementary material for: Influence of oncogenic mutations and tumor microenvironment alterations on extranodal invasion in diffuse large B‐cell lymphoma
Source: Clin Transl Med. 2020 Nov 24;10(7):e221. doi: 10.1002/ctm2.221 (PMC7685246; doi:10.1002/ctm2.221)
Supplement: Supplementary file 4 — Supplementary information TABLE S1 Extranodal involvement of patients evaluated by PET‐CT and CT/MRI [file CTM2-10-e221-s004.docx]

| **Supplementary Table 1** Extranodal involvement of patients evaluated by PET-CT and CT/MRI | | | |
| --- | --- | --- | --- |
| Sites of extranodal invasion | Patients evaluated  by PET-CT | Patients evaluated  by CT/MRI | *P* value |
|  | (N=1382) | (N=578) |  |
| Gastrointestinal tract | 25.9% | 29.9% | 0.0745 |
| Bones | 12.2% | 9.7% | 0.1202 |
| Spleen | 10.9% | 11.2% | 0.8122 |
| Bone marrow | 8.3% | 7.1% | 0.4102 |
| Kidney/Adrenal glands | 5.6% | 5.0% | 0.6629 |
| Lungs | 5.9% | 3.8% | 0.0602 |
| Breasts | 5.2% | 3.8% | 0.2033 |
| Liver | 3.5% | 5.4% | 0.0588 |
| Pancreas | 3.0% | 4.7% | 0.0774 |
| Testes | 3.3% | 3.1% | 0.8896 |
| Central nervous system | 3.1% | 2.6% | 0.6612 |
| Thyroid | 2.6% | 2.2% | 0.7518 |
| Skin | 2.3% | 1.0% | 0.0718 |
| Nasal cavity | 1.7% | 1.4% | 0.8428 |
| Uterus/Ovaries | 1.2% | 0.7% | 0.3458 |
