# Supplementary material for: Influence of oncogenic mutations and tumor microenvironment alterations on extranodal invasion in diffuse large B‐cell lymphoma
Source: Clin Transl Med. 2020 Nov 24;10(7):e221. doi: 10.1002/ctm2.221 (PMC7685246; doi:10.1002/ctm2.221)
Supplement: Supplementary file 5 — Supplementary information [file CTM2-10-e221-s005.pdf]

Wiley Editing Services

ENGLISH EDITING CERTIFICATE

This document certifies that the manuscript listed below was edited for proper English language, grammar, punctuation, spelling, and overall style by one or more of the highly qualified native English speaking editors at Wiley Editing Services

Manuscript title

Influence of oncogenic mutations and tumor microenvironment alterations on extranodal invasion in diffuse large B cell lymphoma

Authors

Rong Shen, Peng-Peng Xu, Nan Wang, Hong-Mei Yi, Lei Dong, Di Fu, Jin-Yan Huang, Heng-Ye Huang, Anne Janin, Shu Cheng, Li Wang, Wei-Li Zhao

Order No

GOZES_1

Date Issued

August 25, 2020

This document certifies that the manuscript listed above was edited for proper English language, grammar, punctuation, spelling, and overall style. Neither the research content nor the authors' intentions were altered in any way during the editing process. Documents receiving this certification should be English-ready for publication; however, the author has the ability to accept or reject our suggestions and changes. If you have any questions or concerns about this document or certification, please contact help-cn@wileyeditingservices.com.

Wiley Publishing Services is a service of Wiley Publishing. Wiley's Scientific, Technical, Medical, and Scholarly (STMS) business serves the world's research and scholarly communities, and is the largest publisher for professional and scholarly societies. Wiley is committed to providing high quality services for researchers. To find out more about Wiley Editing Services, visit http://wileyeditingservices.com. To learn more about our other author services provided by Wiley Publishing, visit https://authorservices.wiley.com/

WILEY
