# Supplementary material for: Influence of oncogenic mutations and tumor microenvironment alterations on extranodal invasion in diffuse large B‐cell lymphoma
Source: Clin Transl Med. 2020 Nov 24;10(7):e221. doi: 10.1002/ctm2.221 (PMC7685246; doi:10.1002/ctm2.221)
Supplement: Supplementary file 6 — Supplementary information Supplementary methods. [file CTM2-10-e221-s006.docx]

**Supplementary methods**

Sequencing and alignment. For whole genome sequencing (WGS), genomic DNA concentrations were measured with the Qubit (Thermo Fisher Scientific), and sheared to about 300bp fragments by Covaris DNA shearing system. After end-repaired and 3’-ends adenylated, Illumina PE adapters were ligated to DNA fragments to generate indexed library. Library was validated by Agilent 2100 Bioanalyzer and sequencing was performed on Illumina HiSeq platform with 150bp paired-end strategy in WuXi NextCODE, Shanghai. For whole exome sequencing (WES), exome regions were captured by a SeqCap EZ Human Exome kit (version 3.0) and sequencing was performed on HiSeq 4000 platform with 150bp paired-end strategy in Righton, Shanghai.

RNA purification, reverse transcription, library construction and sequencing were performed in WuXi NextCODE according to the manufacturer’s instructions (Illumina). PolyA mRNA was purified from total RNA using oligo-dT-attached magnetic beads and then fragmented by fragmentation buffer. The synthesized cDNA was subjected to end-repair, phosphorylation and ‘A’ base addition according to Illumina's library construction protocol. Then Illumina sequencing adapters were added to both size of the cDNA fragments. After PCR amplification for DNA enrichment, the target fragments of 200–300 bp were cleaned up. After library construction, Qubit (Thermo Fisher Scientific) was used to quantify concentration of the resulting sequencing libraries, while the size distribution was analyzed using Agilent BioAnalyzer 2100 (Agilent). After library validation, Illumina cBOT cluster generation system with HiSeq PE Cluster Kits (illumina) was used to generate clusters. Paired-end sequencing was performed using an Illumina HiSeq system following Illumina-provided protocols for 2 × 150 paired-end sequencing.

Read pairs were aligned to Human Reference Genome version hg19 (downloaded from UCSC Genome Browser, URLs) by Burrows–Wheeler Aligner (BWA) version 0.7.13-r1126. Samtools version 1.3 was used to generate chromosomal coordinate-sorted bam files and to remove PCR duplications. The reads were then realigned around potential indel regions by Genome Analysis Toolkit (GATK) version 3.4 IndelRealigner with the recommended pipeline. The mean depth of each sample measured with WES/WGS was 120.25 × (range 50-200 ×), with an average 97.65% (range 82.64%-99.06%) of the target sequence being covered sufficiently deep for variant calling (≥ 10 × coverage).

SNV/indel calling and filter workflow. GATK Haplotype Caller and GATK Unified Genotyper were applied to call SNVs and indels. Homemade pipeline was used to filter SNVs and indels detected by the above software, excluding: 1) mutations reported with low confidence; 2) germline mutations detected from control samples; 3) population-related variants reported in 1000 Genomes (dbSNP 137) as common SNPs and not included in COSMIC (the Catalogue of Somatic Mutations in Cancer) version v77.

SNV/indel annotation. SNVs and indels were mapped to the genome location using the UCSC Genome Browser ([http://genome.ucsc.edu](http://genome.ucsc.edu/)). All the somatic functional mutations, including nonsynonymous SNVs, frameshift or in-frame indels, stopgain or stoploss were obtained. Visual inspection was used to exclude potential false positive results.

Targeted sequencing. PCR primers were designed by Primer 5.0 software. Multiplexed libraries of tagged amplicons from tumor tissue samples were generated by Shanghai Righton Bio-Pharmaceutical Multiplex-PCR Amplification System. Deep sequencing was performed using established Illumina protocols on HiSeq 4000 platform (Illumina).
